# Characterizing the implementation of performance management interventions in a primary health care system: a case study of the Salud Mesoamerica Initiative in El Salvador

**DOI:** 10.1093/heapol/czad020

**Published:** 2023-03-27

**Authors:** L Esther Aranda, Zainab Arif, Cinzia Innocenti, Syed Shabab Wahid, Seble Frehywot, Wolfgang Munar

**Affiliations:** Department of Global Health, Milken Institute School of Public Health, George Washington University, 950 New Hampshire Avenue, NW, Washington, DC 20052, USA; Technical Resources International, 6500 Rock Spring Dr #650, Bethesda, MD 20817, USA; Independent Researcher, Carretera Principal, No 49, Santo Tomas, San Salvador, El Salvador; Department of International Health, School of Health, Georgetown University, 3700 Reservoir Road, NW, Washington, DC 20057, USA; Department of Global Health, Milken Institute School of Public Health, George Washington University, 950 New Hampshire Avenue, NW, Washington, DC 20052, USA; Department of Global Health, Milken Institute School of Public Health, George Washington University, 950 New Hampshire Avenue, NW, Washington, DC 20052, USA

**Keywords:** Primary health care, health systems research, El Salvador, Salud Mesoamerica Initiative

## Abstract

Performance management (PM) reforms have been introduced in health systems worldwide to improve accountability, transparency and learning. However, gaps in evidence exist regarding the ways in which PM contributes to organizational-level outcomes. Between 2015 and 2017, the government of El Salvador and the Salud Mesoamerica Initiative (SMI) introduced team-based PM interventions in the country’s primary health care (PHC) system including target setting, performance measurement, provision of feedback and in-kind incentives. The programme’s evaluation showed widespread improvements in performance for community outreach and service timeliness, quality and utilization. The current study characterizes how the implementation of team-based PM interventions by SMI implementers contributed to PHC system performance improvements. We used a descriptive, single-case study design informed by a programme theory (PT). Data sources included qualitative in-depth interviews and SMI programme documents. We interviewed the members of four PHC teams (*n* = 13), Ministry of Health (MOH) decision makers (*n* = 8) and SMI officials (*n* = 6). Coded data were summarized, and thematic analysis was employed to identify broader categories and patterns. The outcomes chain in the PT was refined based on empirical findings that revealed the convergence of two processes: (1) increased social interactions and relationships among implementers that enhanced communication and created opportunities for social learning and (2) cyclical performance monitoring that generated novel flows of information. These processes contributed to emergent outcomes including the uptake of performance information, altruistic behaviours in service delivery and organizational learning. Through time, the cyclical nature of PM appears to have led to the spread of these behaviours beyond the teams studied here, thus contributing to system-wide effects. Findings illustrate the social nature of implementation processes and describe plausible pathways through which lower-order implementation programme effects can contribute to higher-order changes in system performance.

Key messages

- Performance management interventions are widely used in public management systems around the world, in official development assistance and in the management of primary health care systems in low- and middle-income countries.
- A case study design of an externally evaluated performance management programme in El Salvador serves as the basis for characterizing how programmatic activities, events and implementation processes contributed to the emergence of higher-order organizational and system-level processes of change that contributed to system-wide performance improvements.
- At the organizational level, multi-professional teams engaged in reflection, sense-making and uptake of performance information. At the health system level, programme actors engaged in social processes that increased communications and linkages among them while creating new channels for the dissemination of novel flows of performance information.
- The findings can inform the implementation of public sector reforms in El Salvador and future evaluation efforts of performance management practices in that country. Further research is needed to characterize the role of context in contributing to the improvements in the primary health care system performance observed in El Salvador.

## Introduction

During the last several decades, governments around the world have utilized performance management (PM) systems in public sector reforms to improve organizational effectiveness (Bouckaert and Halligan, 2008; Pollitt, 2013; Van Dooren *et al.*, 2015), including in primary health care (PHC) systems of low- and middle-income countries (Bitton *et al.*, 2019; Munar *et al.*, 2019). Official Development Assistance agencies have also promoted PM strategies in these countries (OECD, 2010) as reflected in the emergence of global health partnerships that have adopted PM to attain performance gains in maternal and child health (MCH), human immunodeficiency virus/acquired immunodeficiency syndrome, tuberculosis and malaria, among other areas.

PM systems have been conceived as ensembles of management control strategies that facilitate the implementation of organizational goals by means of influencing individual and collective behaviours (Broadbent and Laughlin, 2009; Ferreira and Otley, 2009). Examples in the organization and delivery of health services include accountability approaches such as audit and feedback, the public release of performance information and community monitoring of health care providers; implementation strategies such as in-service training, continuous quality improvement and supportive supervision and financial strategies like pay-for-performance and performance-based financing (Munar *et al.*, 2019). When effective, PM systems help managers assess organizational performance and can contribute to the use of performance information to support operational changes, adjust strategic direction and create opportunities for organizational learning (Franco-Santos *et al.*, 2012).

Research evidence on the effectiveness or lack thereof of PM interventions in health systems has accumulated (Björkman-Nyqvist *et al.*, 2017; Munar *et al.*, 2019; Diaconu *et al.*, 2021). A recent evidence gap map summarized the literature on PM systems in the PHC systems of low- and middle-income countries (Munar *et al.*, 2019). It showed that the evidence collected to date had mainly focused on interventions and outcomes at the individual level of providers and patients but had not addressed the organizational and interpersonal levels of analysis. It was also reported that neither the PM interventions nor the primary studies reviewed had used available behavioural and social science theories to conceptualize how PM worked and why, and that few mixed-methods evaluations existed that assessed the ways in which the implementation processes employed by implementers could influence, or not, performance improvement. Given the potential contribution of high-performing PHC systems for the attainment of the Universal Health Coverage agenda (WHO & UNICEF, 2018), the state of art described earlier identified gaps in evidence that need to be addressed.

This paper addresses some of the gaps identified earlier using the experience of the Salud Mesoamerica Initiative (SMI), a multiphase, results-based aid partnership that supported the implementation of health reforms in El Salvador. During Phase 1, the SMI programme in El Salvador was focused on adjusting the policy and regulatory environment for PHC delivery, assessing and improving PM capabilities among Ministry supervisors and team members and piloting teams’ delivery of facility and outreach PHC services (Mokdad *et al.*, 2018). During Phase 2, the SMI introduced the PM interventions mentioned earlier (Bernal and Martinez, 2020). Early results from these measurements showed improvements in the PHC system, prompting us to explore the following research question: How did the implementation of team-based PM interventions by SMI implementers contribute to PHC system improvements in El Salvador between 2015 and 2017? In this paper, we aimed to describe (a) the implementation activities, events and processes employed by SMI implementers in response to the introduction of PM interventions and (b) the outputs and outcomes resulting from programme implementation.

### El Salvador PHC reform

In the two decades since the end of its civil war, El Salvador has improved its human development and health indicators (IADB, 2020; World Bank, 2020). Health outcomes have consistently improved, particularly in MCH, leading to the achievement of the Millennium Development Goal for under-five mortality in 2015, and of the Sustainable Development Goals for maternal and child mortality in 2018 (OHCR, 2017; WHO, 2020).

**Figure 1.**
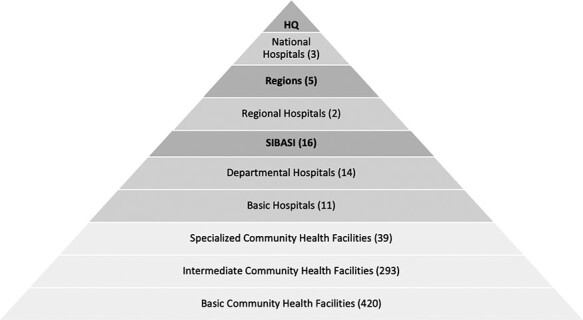
Structure of the health system

In 2009, the government of El Salvador reformed the health system and made access to universal, comprehensive PHC, a right for Salvadorans (Ministerio De Salud, 2019). Health policy and public financing were focused on health promotion and prevention and on the delivery of PHC services to the poorest populations (IADB, 2010; Ministerio De Salud, 2019). Reform implementation led to the reorganization of health facilities into integrated networks of care supervised by departmental-level coordination units, overseen by administrative health regions. In turn, PHC service delivery was delegated to community-based, multi-professional PHC teams (WHO, 2018; Ministerio De Salud, 2019). Figure 1 presents the structure of El Salvador’s health system.

Starting in 2011, and spanning >10 years, consecutive governments in El Salvador have partnered with the SMI seeking to improve the health of women and children among the poorest, hardest-to-reach populations in eight Mesoamerican countries by means of PM strategies that promote the delivery of pre-agreed performance targets among participating governments (Mokdad *et al.*, 2015).

### The SMI programme in El Salvador

The SMI programme supports 75 multi-professional PHC teams located in 14 sites that correspond to the poorest rural municipalities in El Salvador (Gobierno De El Salvador, 2010). As a results-based aid programme, the SMI and the MOH agreed on performance targets, and each contributed half of the funding for the programme. The government is responsible for introducing supportive policies, providing tangible resources such as funding, supplies and training and implementing programme interventions in the targeted areas through its own systems and procedures. If successful in achieving at least 80% of the agreed targets, it receives a performance reward equivalent to half of its funding share (Mokdad *et al.*, 2018). Between 2015 and 2017, the MOH and the SMI implemented supply-side, team-based PM interventions including target setting, performance measurement and provision of feedback and in-kind incentives. Teams’ targets included a set of MCH outcomes grouped into four domains: community outreach, service utilization, timeliness of care and quality of care. Each domain consisted of several targets and corresponding indicators. Community outreach was composed of two indicators: delivery of information on modern family planning and knowledge of treatment of diarrhoea with oral rehydration salts and zinc. Service utilization consisted of four indicators: institutional delivery, micronutrients for children, deworming pills consumption and measles, mumps, and rubella vaccination. Timeliness of care comprised two indicators: timely pre- and post-natal care. Quality of care consisted of two indicators: quality of care according to national clinical guidelines and reference to institutional delivery in birth plans (Bernal and Martinez, 2020). The SMI concurrently conducted an impact evaluation to assess programme effects. Measurement of team performance occurred every six months, consisting of facility and household surveys (Bernal and Martinez, 2020). The final programme evaluation showed significant performance improvements in all four domains. The greatest gains were in community outreach (0.17 SD, *P* < 0.05) and quality of care (0.14 SD, *P* < 0.01). The other two domains had smaller gains: 0.10 SD, *P* < 0.10 for timeliness of care, and 0.096 SD, *P* < 0.05 for utilization (Bernal and Martinez, 2020). The programme evaluation did not describe how such gains were attained or why.

Between 2017 and 2018, and as part of an ongoing realist evaluation (Munar *et al.*, 2018a), we conducted a case study research to explore the behavioural and contextual factors that may have contributed to the gains in performance reported in El Salvador. The study was conducted contemporaneously with the SMI programme and independently from the SMI’s programme evaluation. Enabling factors found to have contributed to high performance included intrinsic motivation among team members, the high level of autonomy delegated to teams by the health reform and the provision of team-specific performance information at learning events attended by the medical coordinators of all teams participating in the SMI.

## Methods

### Study design

We used a descriptive single-case study research design, with a theory-driven approach to data analysis. The case study research has the dual advantage of examining ‘a contemporary phenomenon … within its real world context’ (Yin, 2018) while helping characterize ‘the processes that led to specific outcomes’ (Maxwell, 2012). A descriptive approach is suitable to ‘identify an overall pattern of complexity’ to characterize how reported outcomes were plausibly achieved (Yin, 2018). A single-case design is also appropriate for this study as it may ‘reveal insights about normal processes’ (Yin, 2018). Here, the case is the SMI programme as implemented between 2015 and 2017 in El Salvador.

### Programme theory

Before the data collection started, we built a programme theory (PT) to describe how the effects of the SMI programme, as reported in Bernal and Martinez (2020), were expected to be produced and why. We followed the guidance of previous work on theory-driven evaluation (Chen and Rossi, 1983; Funnell and Rogers, 2011) to integrate the insights from the literature and create the PT. Once built, the PT helped us develop hypothetical propositions (Eisenhardt and Graebner, 2007; Yin, 2018) and provided a testable model of how the outcomes reported in the SMI’s programme evaluation (Bernal and Martinez, 2020) could have been plausibly generated. The social and behavioural science theories that we explicitly considered during the stages of theory design, data analysis, interpretation and organization of findings included diffusion and dissemination theory (Rogers, 2003; Greenhalgh *et al.*, 2004), agency theory (Eisenhardt, 1989), stewardship theory (Davis *et al.*, 1997), self-determination theory (Gagne and Deci, 2005) and goal-setting theory (Locke, 1968). The conceptualization of implementation was guided by the Consolidated Framework for Implementation Research (CFIR) (Damschroder *et al.*, 2009).

**Figure 2.**
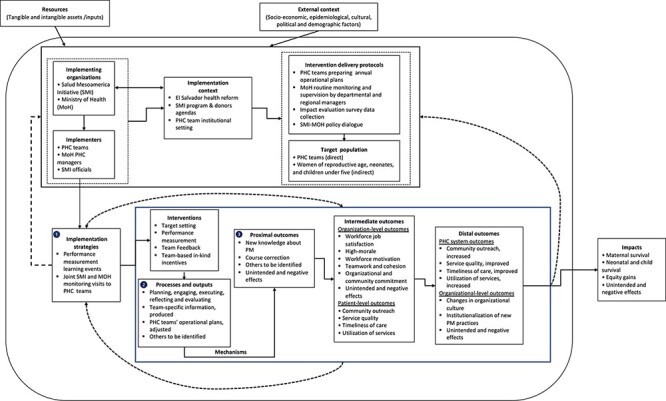
Program theory

The PT contains external and internal component elements that are defined in Table 1 and summarized in Figure 2. The external context and resources are exogenous to the programme but can influence implementers’ behaviours and overall system effects via the barriers and facilitators they can generate. There are three endogenous elements including (1) an action model, containing implementing organizations, implementers, institutional context, intervention delivery protocols and target populations; (2) implementation strategies; and (3) a change model including programme interventions, mechanisms, processes, outputs and outcomes chain. Relationships between elements are shown as solid lines, and dashed lines represent time delays that can create nonlinear relationships and feedback between the elements.

**Table 1.** Elements in the programme theory

Theory elements	Definition	Application to the SMI programme in El Salvador between 2015 and 2017
Resources	The assets or inputs (tangible and intangible) that allow implementing organizations deliver programme interventions and support implementers and/or target populations during implementation.	SMI financial support to the MOH; availability of infrastructure, equipment and supplies for PHC teams to deliver PHC services; time to attend supervision meetings and learning events; MOH-funded training to acquire new clinical or professional skills, etc.
Implementing organizations and implementers	The organizations and individuals that deliver programme intervention services.	Implementing organizations included: (a) the Ministry of Health for the delivery of PM interventions and (b) the SMI. The SMI implementers included the SMI evaluation team and external contractors conducting the verification of performance and the impact evaluation. The MOH implementers included the PHC teams and departmental and regional PHC supervisors.
Implementation context	The ecological setting in which programme activities, events and processes are implemented and in which outputs and outcomes are produced, or not.	Internal setting of the MOH and of each of the PHC teams involved in implementation, including structures, formal and informal rules and norms and the social filters through which people assign meaning. Can favour and/or hinder programme implementation.
Intervention delivery protocols	Formal protocols used by implementing organizations to increase reach of interventions among the target population (Chen, 2015).	Protocols developed by the MOH to conduct routine supervision, to train providers, guidelines or protocols for PHC service delivery, for engaging community organizations, etc.
Target population	The population whose behaviours should change in response to PM interventions.	Team-based interventions targeted all 75 PHC teams and their members. The national-level SMI programme targeted all MOH regional and departmental managers involved in PHC policy making, service monitoring, community engagement and support services (e.g. logistics and supply management, human resources and information management). Women and children were indirectly targeted.
Implementation strategies	The methods or techniques used to enhance the adoption, implementation and sustainability of a programme or practice (Curran *et al.*, 2012)	Activities, events and processes employed by implementing organizations and implementers to deliver the PM intervention and address contextual barriers and facilitators.
Interventions	Team-based performance management strategies introduced to induce improvements in the delivery of health care services.	Target setting, performance measurement, feedback and in-kind incentives.
Processes	Actions or steps implemented to achieve a specific end; work together to produce outputs.	Teams’ internal processes, MOH supervisory activities and field visits.
Outputs	The products of activities, events or processes.	Products and deliverables resulting from programme implementation including data sets, report cards and action plans
Mechanisms	Stakeholder reasoning, values, emotions, norms and collective beliefs that influence the decisions and choices that, in turn, lead to observed outcomes (Hedström and Ylikoski, 2010, Lacouture et al., 2015).	Examples of theory-informed causal mechanisms include, among others, extrinsic motivation (agency theory); satisfaction of basic psychological needs for autonomy, competence and relatedness (self-determination theory); goal commitment (goal-setting theory) and trust and delegation from MOH supervisors to teams (stewardship theory)
Outcomes chain	In the presence of favourable contextual conditions, outputs and mechanisms may contribute to changes in behaviour among those in the target population (e.g. PHC teams and MOH decision makers)	1. Proximal outcomes—immediate changes in attitudes and behaviours among team members as a (direct) consequence of the adoption or uptake of programme outputs, such as changes in motivation, uses of performance information and corrective action to improve service delivery. 2. Intermediate outcomes—changes in teams and team members’ behaviours as direct and/or indirect consequences of proximal outcomes. May include, among others, workforce motivation and job satisfaction, high morale and organizational commitment. Also, community and patient-level health outcomes. 3. Distal outcomes—changes in performance, as reported in SMI’s impact evaluation (Bernal and Martinez, 2020). Also, organizational-level changes in culture, values and collective norms; and the institutionalization and normalization of organizational behaviours and routines. These outcomes are necessary conditions for population health impacts to emerge.

Once we developed the PT, we hypothesized that the implementation strategies used by implementers in the delivery of SMI’s PM interventions would contribute to short-term, processes and outputs and to proximal implementation outcomes. Research has shown that the implementation approaches used to deliver programme interventions and to address contextual barriers and facilitators can lead (or not) to outcomes such as the uptake of new knowledge, or increased programme feasibility, among others (Proctor *et al.*, 2011; Curran *et al.*, 2012). Implementation outcomes can thus influence the occurrence of downstream effects in the outcomes chain and are thus of importance when characterizing the change in complex systems.

In the PT, implementation processes and their immediate outcomes were framed as necessary, albeit insufficient, conditions for the gains in performance reported in El Salvador (Munar *et al.*, 2018b; Bernal and Martinez, 2020). Environmental factors, such as the institutional context of the organizations involved in programme implementation, can also contribute to programme effects.

### Case selection, site selection and sampling

El Salvador’s SMI programme provided a unique and ‘instrumental opportunity’ (Stake, 2008) to explore an issue of interest which in this case corresponds to the activities, events and processes employed by implementation actors. PM interventions were delivered. We first selected 4 of the 14 municipalities in which the SMI was being implemented and then purposively sampled one PHC team from each of the sampled municipalities. The teams that were selected had been categorized by the SMI (Bernal and Martinez, 2020, Bernal (personal communication, October)) as high-performing teams based on their scores from four waves of performance measurement (Table 2). This type of sampling is called conceptual, or deductive theoretical sampling; it was aimed at deepening our understanding of a theory-derived construct (Patton, 2014)—i.e. performance improvement in this case—as defined in our PT, in the social science literature cited earlier, and in the protocol that guided this investigation (Munar *et al.*, 2018a).

**Table 2.** PHC teams’ performance measurement results, 2015–2017

Team	Baseline	6th month	12th month	18th month	Average
1	80.0	90.0	100.0	100.0	92.5
2	81.3	80.0	100.0	95.0	89.1
3	81.3	75.0	95.0	90.0	85.3
4	81.3	70.0	85.0	94.7	82.8

### Data collection and analysis

**Figure 3.**
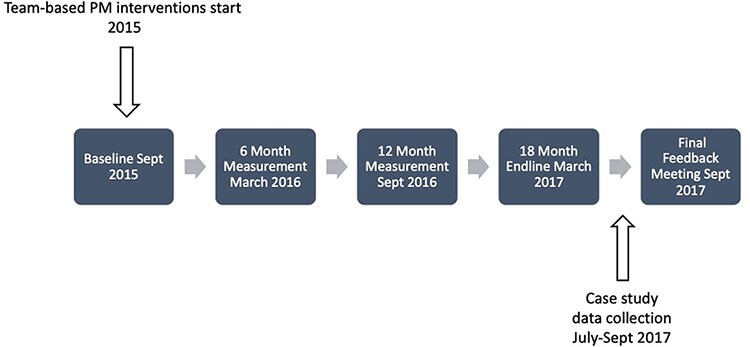
Programme timeline

We used two sources of data including qualitative semi-structured interviews and relevant SMI documents. Criteria for participant selection included prior experience with and knowledge of the El Salvador health reform and direct participation in the implementation of PM interventions. In this way, we favoured respondents’ ability to provide relevant information that could meaningfully contribute to the study’s areas of interest (Pawson and Sridharan, 2010; Malterud *et al.*, 2015). We interviewed 27 respondents, including 13 PHC team members, 8 MOH decision makers and 6 SMI officials. In the teams studied, we interviewed medical coordinators, nurses and health promoters. Interviews were conducted by two researchers between July and September 2017. Figure 3 summarizes the research timeline.

We developed interview guidelines for each type of respondent (Supplementary File S-1). Interviews with team members addressed their professional background, perceptions about programme interventions, perceived barriers and facilitators to teams’ actions, involvement in the SMI’s activities and events, resources and support provided by the MOH and the SMI, and perceived effects from their individual and collective actions. Probes were developed to capture the salience of providers’ experiences in programme implementation and service delivery. MOH and SMI respondents were asked about their perceptions regarding programme interventions, institutional and programmatic context prior to the SMI and the PHC system and teams’ supervision and support. All interviews in El Salvador were conducted in Spanish; a few SMI respondents were interviewed in English, based on their preference. Interviews lasted on average 45 minutes, were recorded with informed consent, transcribed verbatim, professionally translated and imported into NVivo 12. Table 3 summarizes respondent roles and characteristics.

**Table 3.** Respondent roles and characteristics

Stakeholder	Male	Female	Total
1. PHC team members			
• Medical doctor	2	3	5
• Nurse	0	4	4
• Health promoter	2	2	4
2. MOH decision makers	3	5	8
3. SMI officials	2	4	6
Total	9	18	27

We reviewed documents covering several topics including the design of SMI in general and of the El Salvador programme, in particular, SMI programme performance frameworks and implementation plans and MOH policy documents and reports, among others (Supplementary Data S2). This review helped us develop a preliminary understanding of system antecedents, interventions, programme context, implementation approaches and organizations, implementers, target populations, programme resources and the theories of change used in the design of the SMI programme in El Salvador.

We developed a codebook informed by the PT to guide data coding (Supplementary Data S3). Two researchers coded all interview transcripts using deductive and inductive approaches. Code summary memos were then developed to organize the data before conducting thematic, within-case analysis (Yin, 2018; Braun *et al.*, 2019). A code summary memo template is included in Supplementary Data S4. We aggregated within-case themes (Eisenhardt, 2002; Huberman and Miles, 2002) and then identified a reduced number of thematic categories and their relationships (Miles *et al.*, 2018). We used TechEd Marketing’s Inspiration software (https://www.inspiration-at.com) to develop concept maps and causal diagrams to help us make sense of the patterns of relationships identified (Wheeldon and Ahlberg, 2012). We also consulted the literature iteratively as we analysed data to progressively refine the PT and linked the emergent findings to existing behavioural and social science theories (Westhorp, 2013; Yin, 2018). In the final stage, we contrasted the preliminary PT with the findings and refined the outcomes chain.

To increase the credibility of our findings, we used triangulation across data sources (i.e. SMI relevant programme documents and in-depth interviews), respondents, researchers and multiple social and behavioural science theories. The standards for reporting qualitative research checklist is presented in Supplementary Data S5 (O’brien *et al.*, 2014).

## Results

In this section, we first present the implementation approaches reported by respondents including activities, events and implementation processes and then we summarize the outputs and outcomes reported. Activities encompassed teams’ actions or tasks such as facility-based service provision and community outreach services, among other activities. Salient events and implementation processes reported included biannual learning events, quarterly field visits, supervision meetings between MOH decision makers and medical coordinators and a variety of teams’ internal processes and activities (Table 4). These activities, events and processes produced various outputs that, in turn, generated implementation outcomes at the individual and organizational levels.

**Table 4.** Activities, events and processes

SMI implementation approaches	Description
Activities	
Actions or tasks that respondents reported completing individually or in groups	Facility-based service provision: preventive and curative PHC services delivered at the facility. Outreach services provision: health promotion and preventive PHC services delivered in the community, primarily home visits.
Events	
Public meetings, workshops and convenings that took place as part of SMI implementation	Biannual learning events: convened by the MOH and the SMI, attended by all PHC medical coordinators. Performance reports were provided; awards and in-kind incentives were allocated to the high-performing teams, and the most successful teams shared lessons learned. Quarterly field visits by the MOH decision makers and the SMI officials to monitor programme implementation in facilities and in the community. Performance measurement for the SMI impact evaluation: unannounced visits by the SMI impact evaluation team to the PHC teams and allocated households in their area of influence.
Processes	
Actions or steps implemented to achieve a specific end; function together to produce outputs	Teams’ internal processes: reflection, collective sensemaking, performance information uptake and corrective action planning, among others. MOH supervision of PHC teams: routine meetings between regional and departmental MOH managers with medical coordinators to verify and discuss progress.

### Activities and events

Team members were identified, and MOH respondents confirmed, their participation in several types of routine activities and events. These included teams’ delivery of facility-based and community outreach services; routine supervision meetings between MOH decision makers and PHC teams where progress was verified, and challenges discussed; and meetings between the medical coordinators of PHC teams, local leaders and other municipal actors for coordination purposes. High-stakes events were also reported including learning events and field visits.

Biannual learning events were convened by the MOH and the SMI and were attended by the medical coordinators of all teams participating in the SMI. These highly anticipated events served several purposes, including disseminating performance reports, announcing recipients of in-kind incentives and recognition and requiring peer-to-peer knowledge-sharing of best practices. For instance, performance reports from regular monitoring by the SMI were presented and publicly shared; high-performing teams received recognition and in-kind incentives and medical coordinators from the most successful teams shared lessons learned during implementation with peers. Medical coordinators reported sharing the feedback and performance reports received in the meetings with their entire teams upon returning to their communities to begin planning for corrective actions.

Quarterly field visits were also conducted by SMI officials and MOH decision makers to monitor programme implementation at PHC facilities and in the community as teams delivered their services and conducted outreach activities. During these visits, team members such as medical coordinators, nurses and health promoters reported their progress and discussed implementation challenges and bottlenecks. Finally, unannounced visits took place every six months by the SMI monitoring team to collect facility and household survey data that were then summarized and fed back to the MOH for performance monitoring purposes and ultimately disseminated to medical coordinators at the biannual learning events mentioned earlier.

### Implementation processes

Several formal and informal processes were deployed by implementers during programme implementation including (1) performance target-setting and communication, (2) emergent problem-solving and learning spaces and (3) reflecting on progress, making sense of performance information, and evaluating future actions.

#### Performance target-setting and communication

Team members reported that their performance targets were established for them by the MOH, were focused on national MCH priorities and were reflected in team-specific annual operational plans. According to SMI respondents, targets served as a means for the MOH to hold teams accountable for results and to determine eligibility for in-kind incentives and awards. Team members perceived value in having access to clearly articulated, precisely framed and well-communicated performance targets. Targets were reported as providing teams focus and a sense of direction not previously experienced.

*Before we did not have achievable goals or objectives. Sometimes you sort of get the grasp of things, but when you know that there is a goal to achieve, you make a bigger effort, and you schedule yourself better. You have more optimism when you know you have gone up and you do not want to lose that level* (health promoter).

#### Emergent problem-solving and learning spaces

Implementers reported adapting some of their internal routines in response to the PM interventions. For instance, MOH decision makers adjusted routinely scheduled supervision meetings with teams to discuss progress towards the achievement of targets. Medical coordinators reported MOH and SMI field visits that were seen by them as opportunities to not only report progress towards target achievement but to also share challenges and persistent bottlenecks such as insufficient resource availability, poor transportation and security concerns. Solutions to teams’ challenges were oftentimes identified during these interactions.

*We didn’t have [Intra-Uterine Device] insertion equipment. So, they asked for it and, at that moment, the coordinator of services provision approved it. And, in that [same] week, they had the IUD insertion equipment* (MOH decision maker).

Medical coordinators also reported regularly scheduled opportunities for learning and knowledge-sharing such as the biannual learning events jointly convened by the MOH and the SMI. The extensive participation of all 75 teams in the SMI area of influence was the result of a decision by the Minister of Health who wanted all teams to benefit from what was perceived to be a positive learning and knowledge-sharing experience. In these convenings, medical coordinators networked with their peers from other SMI sites and allowed medical coordinators of high-performing teams to share how they implemented their activities and attained such levels of performance. Although some MOH decision makers perceived these activities to be a form of benchmarking, medical coordinators consistently framed them as opportunities to learn from and help their peers:

*[When] I go to the meeting, I see this more as an opportunity for sharing my experience with my peers. I don’t see that we compare ourselves to others. It’s more about the things we are doing well. The things they can do well. And what we can do well by learning from other peers* (medical coordinator).

All team members interviewed reported anxiety as the date for learning events approached. This was partly due to the high stakes involved in being evaluated and receiving feedback on their performance; also, to the public nature of these events and to the attendance of the MOH and SMI leadership. Despite this anticipatory anxiety, all team members reported feelings of joy and pride when they learned they had achieved or exceeded the threshold for eligibility for in-kind incentives and received certificates of recognition for their efforts.

*Eventually those results show whether we have achieved the objectives; if we are working well; if we have to do more or continue improving, So, all that, eventually serves a purpose which is letting you know how you are really working. And that helps you improve, right?* (nurse)

#### Reflecting on progress, making sense of performance information and evaluating future actions

After attending learning events, medical coordinators shared the scores and feedback with their teams, reviewing the performance information obtained, and engaging in internal processes of analysis to assess the operational implications for course correction and future action. The latter were reflected in informal brainstorming exercises and adjustments to operational plans that were largely focused on increased outreach activities.

Respondents reported that field visits by the SMI and the MOH provided additional opportunities for learning and knowledge-sharing. During these visits, MOH decision makers and SMI officials would directly accompany teams in the delivery of routine activities. SMI officials reported using these visits to explore teams’ capability to interpret performance information and data from the routine health information system. Team members and MOH decision makers reported finding these data helpful in gauging their progress, conceptualizing root causes for potential underperformance and, in general, serving as an early warning system of value to implementers.

### Outputs and performance outcomes

Several outputs were reported from the implementation processes described earlier (Table 5), including performance information reports originated from the survey data used to monitor teams’ performance, the routine health information used by the MOH for teams’ supervision, improvement plans developed by implementers in response to feedback and access to performance information and operational innovations generated by some teams such as community enumeration tools to help with planning community outreach visits or dashboards developed by the MOH to visualize teams’ progress and early identification of challenges. Some of these tools spread across the PHC system during learning events while others, like the dashboards, diffused beyond El Salvador.

**Table 5.** Programme outputs

Outputs	Representative quotes
Performance information reports	*[We] did the missions on a quarterly basis for the evaluation. In one quarter we had external monitoring results [from SMI], and in the other results from the MOH information systems for follow-up purposes* (MOH decision maker). *And when the doctor says, you got, for example in the last one, 85 points…we felt happy and proud, together as a team, because we work as a team* (health promoter).
Improvement plans	*As I’m telling you, sometimes it makes us a bit sad when we see we didn’t achieve the goal. But at the same time, that’s what drives us to improve and to seek new strategies to see how we can overcome that* … (nurse). *You see, as a team we sat down and started to try and see why [a low score] happened…And we did it among all of us, the team, we look for the reasons and we found the reason why. And that’s how we managed to improve previous results* (health promoter).
Operational innovations and service delivery tools	*I invented these maps here! Like, how to score kids, women of reproductive age. [That way] we knew that if we went to an area, we would have this number of kids, women in fertile age, and pregnant women. And what did we do? Make the most of the time we had for the visit* (medical coordinator).

Several organizational-level outcomes emerged during the study period (Table 6) including collective uptake and sensemaking of novel performance information by teams and MOH decision makers that reportedly helped them reflect upon plausible root causes for underperformance and bottlenecks, identification of early signs of underperformance and planning improvement actions; new team management practices such as debriefs and planning meetings; learning outcomes in the form of implemented improvement plans that tested corrective actions in response to performance information; altruistic team behaviours including working during weekends and using personal funds to reach distant sites where increasing community outreach could address performance gaps; and unintended outcomes such as increased workload, anxiety or low morale when attaining lower-than-expected performance results.

**Table 6.** Performance outcomes reported

Outcomes	Representative quotes
Uptake of performance information and reflective practices	*[Performance information] helps us because we need a way of having a parameter to know if we are on the right track or if we are completely lost. That is where we make modifications. And if we did wrong, we must change it. We can improve those situations* (medical coordinator).
Improvements in service delivery	*[Learning events] and peer exchanges are really helpful. Eventually, I mean, you take [from them] what you think can be useful to improve, right?* (nurse). *No, we reorganize the work. The duties don’t change, but…the teamwork was essential to be able to carry out our activities and to organize the planning* (medical coordinator).
Altruistic behaviours	*We don’t have an ambulance right now, so [the driver] charged us forty dollars to take us there…Sometimes the whole team pays for [transportation], and other times we do the following: we collect paper, useless sheets of paper, and the person from the file sells them. That money makes a fund, and we collect it in a box* (nurse). *Yes, it’s a [cell phone] plan we pay additionally. In that way, we are always connected, we are in communication day and night with our partners* (health promoter). *As I say, our objective is that we are healthcare workers, and in community health, we say that we are 24/7, like the ATM machines, you know? Because we don’t have a work shift* (health promoter).
Unintended outcomes	*And when we get good scores, we feel motivated, and to get low scores, you bet, it demotivates us, too, at the time* (health promoter).

## Discussion

**Figure 4.**
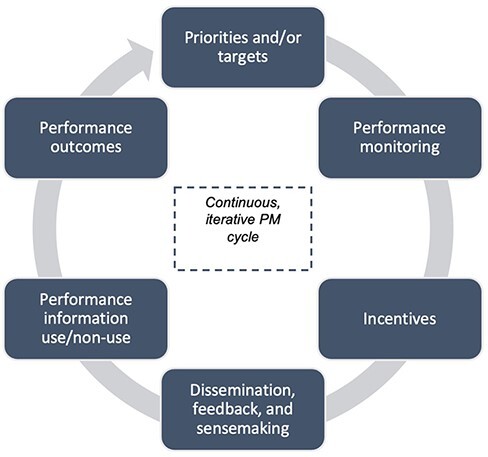
The structure of the PM system

In this case study, we characterized the ways in which SMI activities, events and implementation processes and their related outputs and outcomes appear to have contributed to the widespread improvements in PHC system performance reported in El Salvador between 2015 and 2017 (Bernal and Martinez, 2020). Our findings indicate that during the period of study, both the implementation approaches and the PM interventions introduced into the PHC system contributed to the emergence of a functioning PM system. The latter showed structural components similar to the ones conventionally described in public management literature in industrialized nations (Pollitt, 2018). The component elements identified operated in a cyclical way and included priorities, performance measurement, incentives, dissemination and feedback, performance information use and performance outcomes. The emergent PM system and its component elements are depicted in Figure 4.

Some elements of this emergent PM system existed before the SMI started. For instance, policy priorities for the universalization of PHC services had been set by the MOH; and governance structures and routine managerial practices existed for overseeing and supervising PHC service delivery. On the other hand, the SMI introduced novel interventions such as in-kind incentives and measurement of teams’ performance using household and facility surveys. The use of surveys appears to have been driven not only by conventional monitoring and evaluation practices used by development programmes but also by the need to collect data for the programme evaluation that took place concurrently with implementation.

Other elements in this novel PM system appear to have emerged organically as lower-order actions, and decisions by implementers led to the outputs described earlier. In turn, the effects of these decisions and the cyclical repetition of PM practices over 2 years of implementation seem to have contributed to higher-order outcomes that, in turn, led to the emergence of third-order sociotechnical processes, such as the dissemination of knowledge and evidence across the PHC system, which helped hold together the emergent PM system. The micro-level decisions by implementers reported here included, among others, engaging the entire population of PHC teams in biannual learning events, promoting peer-to-peer knowledge sharing during those events, conducting MOH and SMI joint field visits to observe the work of teams and regularly feeding teams with actionable and specific performance information. Decisions made by teams in response to such information included, among others, the introduction of operational innovations, processes of collective reflection and sense-making, uptake and use of performance information for planning improvement actions and, ultimately, the implementation of improvement plans that contributed to the team-level performance outcomes reported here.

**Figure 5.**
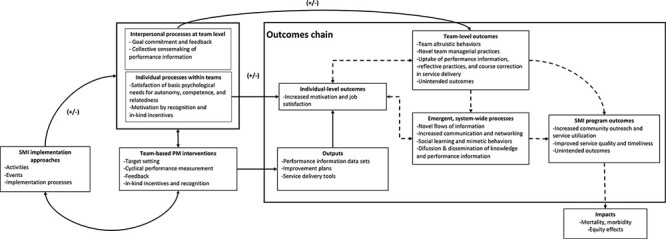
Refined outcomes chain

However, moving from individual and interpersonal processes of change to the PHC system improvement reported by Bernal and Martinez (2020) requires causally connecting individual decisions and team-based behaviour to the wider PHC system. We identified two emergent, higher-order processes of this nature. First, the emergence of new flows of performance information that were widely disseminated across all PHC teams in SMI programme sites. These appear to have contributed to performance outcomes through collective behaviours such as the purposive use of performance information to inform planning and support the introduction of operational improvements and course correction, ultimately contributing to organizational learning. Second, our findings show a complementary expansion of formal and informal channels of communication among implementers that not only increased social interactions among them but also appears to have helped as a channel of diffusion and dissemination of information and knowledge across the PHC system. Based on the multilevel process of change described here, we refined the outcomes chain in our PT as indicated in Figure 5.

We also identified contextual factors that appear to have contributed to programme effects. For instance, the use of performance information among teams appears to have emerged from interpersonal processes such as the sharing of lessons during learning events. Informal, peer-to-peer networking seems to have also contributed to the dissemination of practical knowledge about ‘how’ high-performing teams achieved their performance targets. When presented as action-oriented information, the dissemination of practical knowledge in social spaces such as the ones described here, might not only helped diffuse information through formal and informal channels but may have also helped turn knowledge into action (Dougherty, 2004) and influenced attitudes and behaviours among multiple actors in the health system (Rogers, 2003; Greenhalgh *et al.*, 2008).

Participation in learning events, while limited to medical coordinators, was reported by all team members as a high-stakes, salient event for the entire team. After attending, medical coordinators engaged their entire teams in organic processes of debriefing and reflection that seemed to have helped in the collective sensemaking of the performance information and feedback acquired. This seemingly helped teams plan future collective action in the form of increased community outreach services.

The findings also indicate that end-users mainly utilized performance information for instrumental purposes. In evaluation studies, this refers to the use of evidence for decision-making (Alkin and King, 2017). Instrumental use was exemplified in our findings by teams’ decisions to utilize performance information to adjust service delivery, by MOH decision makers to fine-tune the frequency of their supervisory practices and by SMI officials to define how to present and summarize raw performance data to MOH actors and teams alike. Respondents also described a high degree of alignment between the targets used by the SMI to measure their performance and their own internal goals, values and preferences. Targets that linked teams’ performance to the community’s health had profound appeal for respondents. This may have increased the perceived value of the provision of team-specific feedback in a continuous fashion, particularly as such information was perceived to be credible and trustworthy while also confirming providers’ perceptions of their impact on communities’ health. These findings are supported by previous studies on goal-setting theory (Locke and Latham, 2002; Latham *et al.*, 2008) and feedback intervention theory (Kluger and Denisi, 1996).

Other theories not considered in the design of our PT could account for how implementation processes contributed to system-wide change. For instance, the literature on health care decentralization highlights the concept of decision space as the range of choices allowed by the central level to local managers (Bossert, 1998), which might be relevant here, given findings indicating that despite lack of resources, logistical challenges, work-life imbalance and security concerns, the teams in our sample prioritized community outreach and home visits as the main tactics to improve performance. Another alternative explanation to some of our findings could be that collective norms may have promoted cohesion and collaborative teamwork, independently of, or in conjunction with, the other propositions contained in our PT. For instance, within-team collaborative action appears to have provided team members with a sense of satisfaction and pride, an intrinsic motivator that seems to have been positively reinforced by increased access to performance information. The adherence to existing collective norms may also reflect a sense of organizational identity among PHC teams, as reported in some sociological theories of the organization (Ashforth *et al.*, 2011; Salas *et al.*, 2018; Rapp and Mathieu, 2019; Van Wijk *et al.*, 2019).

The processes of improvement and organizational learning reported here and expressed in course correction and iterative implementation of improvement actions contrasts with the conventional views of PM from an agency theory perspective (Eisenhardt, 1989). According to such views, PM mainly operates as a system of behavioural control and hierarchical accountability (Ferreira and Otley, 2009; Franco-Santos *et al.*, 2012). Yet, our findings are more in line with stewardship perspectives in organizational science (Davis *et al.*, 1997) that highlight the role of trust in managerial processes, and with views of health systems as open, social systems (Newton-Lewis *et al.*, 2021).

This study has several strengths. The use of a PT not only guided data analysis but also ensured that the findings contributed to the creation of new knowledge to complement existing social and organizational science theory (Maxwell, 2012) and to identify additional research needs. Considering rival explanations that could have accounted for the reported results increases the validity of case study research (Yin, 2018). To enhance research quality, we triangulated methods, respondent accounts, researcher interpretations and theories (Denzin and Lincoln, 2011). Our study also has some limitations. The use of a single-case study design limits generalizability to other contexts (Yin, 2018) or other populations of PHC providers. Future comparative case study research conducted in different contexts and within wider populations of providers is recommended to increase generalizability. The potential may exist for social desirability bias as reported in qualitative research in general (Johnson and Van de Vijver, 2003), but the use of triangulation across respondents and data sources increases the credibility of our interpretations.

## Conclusion

The findings presented here contribute to the small but growing literature addressing the use of performance information in the context of public sector reforms (Van Dooren and Van De Walle, 2008; Moynihan and Pandey, 2010; Pandey, 2015). To the best of our knowledge, there are no prior case studies addressing the contributions of implementation of PM interventions in the context of health reforms in low- and middle-income countries, nor of the integration of a determinant framework like CFIR in theory-driven evaluation. There are also limited examples of the successful integration of PM and programme evaluation processes. A study of public agencies in the USA that integrated programme evaluation and routine performance data showed that when the two converge, it can ‘facilitate performance information use by reducing the causal uncertainty that managers face as they try to make sense of what performance data mean’ (Kroll and Moynihan, 2018). Our data suggest that PHC teams and MOH decision makers in El Salvador may have organically integrated these two types of evidence as part of the activities, events and social interactions described in this study. Findings also suggest that the exposure of medical coordinators and MOH decision makers to evaluation evidence may not only have increased credibility and trustworthiness in an external source of evidence but that the exposure to new flows of performance information may have served as a tipping point (Meadows, 1999) that shifted system behaviour towards higher levels of aggregate performance.

Our findings reinforce the realist view of social programmes as ‘undeniably, unequivocally, unexceptionally social systems’ (Pawson and Tilley, 1997). During the period studied here, the engagement of implementers in continuous processes of networking, learning and knowledge-sharing appear to have converged in ways that enabled the emergence of a PM system that not only improved accountability and managerial control over the production of PHC services but also contributed to PHC teams’ autonomy, and to the widespread use of performance information for improving service delivery. Ensuring the maintenance of both the structural elements as well as the social processes of the PM system that emerged during implementation seems to be necessary conditions for sustaining PHC system performance through time.

While we identified contextual factors with plausible causal influence on PHC performance, a richer understanding of the process of change that took place in El Salvador during the study period would require detailed characterization of the implementation context. In turn, this would demand the characterization of several environmental factors including, the inner setting in which individual programme actors’ are embedded and of its resources and reasonings (Pawson and Tilley, 1997), the interpersonal linkages connecting implementing actors, their collective rules and social norms (Greenhalgh and Manzano, 2022) and the health system’s broader social, economic, cultural and political settings (Pawson, 2013).

As the SMI winds down, it is advisable to conduct a summative evaluation of the decade-long activities implemented in El Salvador to attain a longitudinal and more nuanced understanding of what worked, how, for whom and why. Additional research is also needed to better characterize the role of context in explaining the results reported here. Finally, it is advisable to study the resilience of the novel PM system described here in response to changes in context such as the introduction of SMI’s third and final phase, the inauguration of a new administration in 2019 and the external shocks brought about by the respiratory infectious disease caused by the SARS-CoV-2 virus (COVID-19) pandemic.

## Supplementary Material

czad020_Supp

## Data Availability

Data are available upon reasonable request. Deidentified transcripts are available from the corresponding author on request.

## Reflexivity statement

The authors of the paper include four females and two males and span a wide range of seniority and experience. Their research experience spans low- and middle-income countries in Africa, Asia and Latin America and the Caribbean. Four authors have extensive experience conducting qualitative and mixed-methods health policy and systems research in areas such as performance improvement, organizational change, global workforce, equity, global mental health and maternal and child health. The authors who collected qualitative data have extensive experience conducting qualitative fieldwork in El Salvador and other low- and middle-income countries. Additional background and experience for each author is provided in Supplementary Data S6.
