# Supplementary material for: Characterizing the implementation of performance management interventions in a primary health care system: a case study of the Salud Mesoamerica Initiative in El Salvador
Source: Health Policy Plan. 2023 Mar 27;38(5):579–92. doi: 10.1093/heapol/czad020 (PMC10190960; doi:10.1093/heapol/czad020)
Supplement: czad020_Supp [file czad020_supp.zip › S-1 Interview guidelines.pdf]

SUPPLEMENTARY FILE S-1 – INTERVIEW GUIDELINES-EL SALVADOR

1.1 TEAM MEMBERS INTERVIEW GUIDELINE

1. In 2011, where were you working? What was your role? *(Probe roles and responsibilities in the team and the Salud Mesoamerica Initiative (SMI)*
2. What are your roles and responsibilities in your current job?
3. What are the most common health issues in your municipality? *(Probe about health maternal and neonatal issues)*
4. What do you like the most about SMI? How has it helped?
5. As you know, SMI conducts performance evaluations; Your team has been positively assessed. How does it feel to have done well in the evaluation?
6. Let's further explore this "feeling good" in the evaluation. Why does it feel good when the team demonstrates good performance or comes out "well evaluated"?
7. What do <u>you</u> feel when the result from an evaluation is favorable?
    a. Why?
8. In your team and in this health center many individuals work. What happens when an individual does not do his/her job well?
    a. What does the group do?
    b. Who takes care about these matters?
    c. What have you all learned from these situations?
    d. (*For each source of motivation mentioned*) I want to better understand that idea you just mentioned. Why is it produced? How does it feel when you do "that"?
9. What effects do you think that are produced from receiving *community recognition*?
10. Let's discuss compensation now *(salary, payment, bonuses)* of this team/health center's staff. How do you feel about such compensation? Does it truly represent the work done? How important is to feel that the salary recognizes the work? Why? *Give me an example*.
11. Some say that if one gives a bonus or better compensation, such a thing could reduce the internal drive for work. What do you think about this?
12. Folk seem to give importance to the regional meetings where you all discuss the results from the team/Health Center. Have you attended such meetings? What do you think about them? What is the use of attending those meetings?
13. Sometimes comparisons between teams with different performance are done in these meetings. Have you been to meetings where these comparisons are made? What do you think about that? What is the use for you all from attending these kinds of meetings? *(Probe the feelings and reactions from participating in benchmarking exercises).*
14. Have you participated in any of the Collaboratives? What has been your experience? (*Probe for motivation*)
    a. How does it feel to participate?
    b. What do you feel?
    c. What is the use of these collaboratives?

1.2 MINISTRY OF HEALTH AND SALUD MESOAMERICA INITIATIVE INTERVIEW GUIDELINE

Let's start by exploring the beginning of the Salud Mesoamerica Initiative (SMI). Tell me about the pre-existing political, policy, and programmatic conditions that led countries (or a specific country) to the decision of participating in SMI?

Probes:

- What role did government priorities in played?
- Did the Central American integration structures play any role? What/how?
- What influence did international/global commitments play? Why? Can you describe it? (Probe for the Millennium Development Goals)
- What role, if any, did the involvement by the Bill & Melinda Gates Foundation and the Carlos Slim Foundation played in the government's decision? Why? Please describe.
- How relevant do you think was the involvement by the Inter-American Development Bank?
- Any other factors in the environment that made SMI attractive to the governments?

Let's discuss the results-based financing approach used in SMI.

- What aspects of this approach are appropriate?
  - ✓ Why?
- How about negative aspects?
  - ✓ Why?
- What are the effects of this results-based financing approach for the ministries of health?
  - ✓ (If applicable) For the regional levels?
  - ✓ For the local levels?

Now let's discuss the data collected through the external verification of performance (EVOP).

- How are EVOP results received once they are presented to country actors?
- What actions are taken by the government?
- What aspects of the EVOP are attractive for the ministries? Anything worrying?
  - ✓ What is the role played by the Inter-American Development Bank in the EVOP process?
- What aspects of the EVOP approach are appropriate?
  - ✓ Why?
- How about negative aspects?
  - ✓ Why?
- What are the effects of the EVOP for the central level of the Ministry of Health (MOH)?
  - ✓ (If applicable) For the regional levels?
  - ✓ For the local levels?

- ✓ How are these results disseminated?
- Is the MOH staff prepared to use the resulting EVOP data?
- What does the central level do with the results? Regional levels (if applicable)? Local levels?
- What is the value-added for the MOH from this EVOP?
- What else could SMI do in the future to support the ministries for better using the EVOP data?

Let's talk about the future, once SMI ends.

- Do you think the EVOP needs to continue once the Initiative ends? Why?
- What options are there for the continuation of the EVOP?

If an external verification weren't possible, should the MOH develop internal verification mechanisms? What would be the obstacles or challenges for this option?
