# Supplementary material for: Characterizing the implementation of performance management interventions in a primary health care system: a case study of the Salud Mesoamerica Initiative in El Salvador
Source: Health Policy Plan. 2023 Mar 27;38(5):579–92. doi: 10.1093/heapol/czad020 (PMC10190960; doi:10.1093/heapol/czad020)
Supplement: czad020_Supp [file czad020_supp.zip › S-2 Document review list.pdf]

Supplementary File S-2 Document review list

PHC Performance Management – El Salvador Case Study Document Review

**General instructions: Fill out information for each document reviewed**	Date created: July 7, 2021<br>Date modified: June 22, 2022

Doc No.	Author	Year	Title	Document Type	Brief description of the document.	Reviewer Initials (removed for peer review)	Notes Also indicates most recent date accessed.
1	Ministry of Health of El Salvador	2009	Informe de labores 2008-2009 (June1, 2008-May 31, 2009)	Report	This document describes the MOH accomplishments, including by regions. It includes an organization chart and lists all the guides/guidelines that were issued during the year. Prior to the 2009 reform, there were 3 national and 5 regional hospitals and 17 SIBASIs responsible for the regional and general hospitals and health units, rural centers, and health homes.		Good document to get a sense of the context before the reform and SMI program.<br><br>Accessed September 24, 2021 https://w5.salud.gob.sv/archivos/pdf/Informes-Labores-MSPAS-MINSAL-2004-2015/INFORME-LABORES-MSPAS-2008-2009.pdf
2	Ministry of Health of El Salvador	2010	Informe de labores 2009-2010	Report	The health reform, published in the Diario Oficial on February 17, 2010, reorganized the health system. An org chart is included in this report. The MOH clearly establishes women and children's health as priorities. The MOH identified the lack of a human resources development policy and set out to establish one where staff receives appropriate training, resources, and continuing education. The MOH is working on developing one health information system that can provide actionable information. This health system is partly funded by IADB.		The MOH is focusing efforts to strengthen the workforce, including improving its HMIS.<br><br>Accessed September 24, 2021 https://w5.salud.gob.sv/archivos/pdf/Informes-Labores-MSPAS-MINSAL-2004-2015/INFORME-LABORES-MSPAS-2009-2010.pdf

Doc No.	Author	Year	Title	Document Type	Brief description of the document.	Reviewer Initials (removed for peer review)	Notes Also indicates most recent date accessed.
3	Ministry of Health of El Salvador	2010	Política nacional de salud, 2009-2014. Construyendo la esperanza. Strategias y recomendaciones 2009-2014.	Policy document	The health reform was published in the Diario Oficial on February 17, 2010. The Ley del Sistema Nacional de Salud (2009) created the integrated health system, based on a family service model. The health reform is based on the principles of equity, commitment, intersectoriality, universality, fee-free service, solidarity, transparency, and social participation. This document describes the (25) strategies to implement the new national health system based on health as a public good and human right.		Although the health reform was officially published in 2010, the new administration immediately set out to develop the reform when it came into power in June 2009.<br><br>Accessed October 26, 2021 https://w5.salud.gob.sv/archivos/pdf/PoliticaNacSalud2009-2014/Pol%C3%ADtica+de+Salud+-+Construyendo+la+Esperanza.pdf
4	Ministry of Health of El Salvador	2011	Informe de labores 2010-2011	Report	The Asamblea Legislativa approved in 2010 a 60M loan from the IADB to fund implementation of the integrated health system in three departments and San Salvador metro area, known as the Programa Integrado de Salud (PRIDES). Also, this report is the first to mention the Salud Mesoamerica working in 14 municipalities.		The PRIDES loan supported implementation of the health reform in three Departments and the San Salvador metro area. It focused on the reform "hardware".<br><br>Accessed September 24, 2021 https://w5.salud.gob.sv/archivos/pdf/Informes-Labores-MSPAS-MINSAL-2004-2015/INFORME-LABORES-MSPAS-2010-2011.pdf

Doc No.	Author	Year	Title	Document Type	Brief description of the document.	Reviewer Initials (removed for peer review)	Notes<br>Also indicates most recent date accessed.
5	Ministry of Health of El Salvador	2011	Curso del promotor de salud. Cuaderno de trabajo.	Other	This workshop workbook introduces the health promoter to the health reform policy; PHC values based on leadership, empathy, quality care, and warmth towards users; PHC emphasis on health promotion and prevention, listing tasks; the PHC team functions, noting that teams (ECOS) were deployed first in the poorest municipalities (p.31); and supervision by specialized teams, SIBASI, Technical Regional Team, and Central team. The document describes job functions and tools such as the community health map. It also lists qualities and skills promoters must have, including leadership skills, social commitment, and willingness to serve. Promoters organize the community health committee. The Community Assembly helps select the health promoter and supports development of an annual operations plan and its evaluation every six months.		The guidance from the MOH includes mention of leadership, empathy, quality of care. The MOH was seeking promoters with certain characteristics such as "leadership skills, social commitment, and willingness to serve," as exemplified by the respondents interviewed.<br><br>Accessed October 19, 2021<br>https://rrhh.salud.gob.sv/files/webfiles/cursos/cuaderno_promotores_de_salud.pdf
6	Ministry of Health of El Salvador	2012	Informe de labores 2011-2012	Report	A loan from IADB funded the renovation of 18 health facilities [Programa Integrado de Salud (PRIDES)]. SMI's first operation is listed in the budget.		Accessed September 24, 2021<br>https://w5.salud.gob.sv/archivos/pdf/Informes-Labores-MSPAS-MINSAL-2004-2015/INFORME-LABORES-MINSAL-2011-2012.pdf
7	Inter-American Development Bank	2012	Convenio de financiamiento no reembolsable del Fondo Mesoamericano de Salud entre la Republica de El Salvador y el Banco Interamericano de Desararollo.	Other	This agreement lays out the first operation of SMI in El Salvador. It also provides background information on the health context.		Accessed October 28, 2021<br>https://www.iadb.org/projects/document/EZSHARE-2056220512-3767?project=ES-G1001;GRT/HE-12982-ES;GRT/HE-12983-ES

Doc No.	Author	Year	Title	Document Type	Brief description of the document.	Reviewer Initials (removed for peer review)	Notes Also indicates most recent date accessed.
			Programa Salud Mesoamerica 2015 - El Salvador				
8	Ministry of Health of El Salvador	2013	Informe de labores 2012-2013	Report	This report provides a good summary of the PRIDES loan from IADB. For the work year, PRIDES loan funded 436 hires to form 45 PHC teams. Funds were also used for information infrastructure. SMI funded hiring and training personnel; acquiring supplies, medicines, equipment, and vehicles.		Accessed September 24, 2021 https://w5.salud.gob.sv/archivos/pdf/Informes-Labores-MSPAS-MINSAL-2004-2015/INFORME-LABORES-MINSAL-2012-2013.pdf
9	Ministry of Health of El Salvador	2013	Manual de organización y funciones de las redes integrales e integradas de servicios de salud	Other	This manual describes each component of the RIIS (health system network). It establishes the meeting schedule as follows: micro network-monthly, department-monthly, regional bimonthly, and national-quarterly (see pg. 26 for participants). There are 11 basic hospitals, 14 Department hospitals, 2 Regional hospitals (E-W), and 3 specialized hospitals. Includes templates for agenda, "acta" meeting summary, and improvement actions.		The MOH established a schedule for various meetings at the different levels of the health system. This administrative practice is highly relevant to our study.<br><br>Accessed October 19, 2021 http://asp.salud.gob.sv/regulacion/pdf/manual/Manual_de_Organizacion_y_Funciones_de_las_RIISS.pdf
10	Inter-American Development Bank	2014	Convenio Individual de Financiamiento No Reembolsable de Inversión del Fondo Mesoamericano de Salud GRT/HE-12982-ES, GRT/HE-12983-ES. Primera Operación	Other	Modification to first SMI agreement. Clarified when the performance tranche would be disbursed. Also made clear the procedures for moving forward with the first disbursement for the second operation, conditional on the minimum achievement of performance levels.		Accessed October 28, 2021 https://www.iadb.org/projects/document/EZSHARE-2056220512-731?project=ES-G1001;GRT/HE-12982-ES;GRT/HE-12983-ES

Doc No.	Author	Year	Title	Document Type	Brief description of the document.	Reviewer Initials (removed for peer review)	Notes<br>Also indicates most recent date accessed.
			Individual. Modificación No. 1.				
11	Ministry of Health of El Salvador	2014	Informe de labores 2013-2014	Report	One of MINSAL's goals is to continue to implement SMI. Funds were also used to develop the SUIS (HIS), including mapping facilities with GIS.		SMI supported the development and improvement of the HMIS.<br><br>Accessed September 24, 2021 https://w5.salud.gob.sv/archivos/pdf/Informes-Labores-MSPAS-MINSAL-2004-2015/INFORME-LABORES-MINSAL-2013-2014.pdf
12	Ministry of Health of El Salvador	2014	Lineamientos tecnicos para la supervision de enfermeria en las riiss	Guideline	This document describes the role and responsibilities for nurse supervision by level. It includes over 20 qualities a supervisor should have, including warmth, leadership, empathy, and equity.		Guidance from the national level emphasizes leadership and empathy, among qualities of PHC supervisors.<br><br>Accessed October 19, 2021 http://asp.salud.gob.sv/regulacion/pdf/lineamientos/lineamientos_tecnicos_supervision_enfermeria_riiss_31012014.pdf
13	Ministry of Health of El Salvador	2014	Resultados del taller de profundizacion de la reforma de salud de El Salvador	Report	The MOH (Dra. Violeta Menjívar) presented accomplishments in each of the eight axes of the reform (2 more added in 2015). These were also documented in the annual informe laboral. The workshop sought to get input for the next planning cycle.		Accessed August 10, 2021<br><br>https://w3.salud.gob.sv/download/sistematizacion-del-taller-de-profundizacion-del-proceso-de-la-reforma-de-salud-en-el-salvador-julio-2014/
14	Ministry of Health of El Salvador	2015	Informe de labores 2014-2015	Report	One goal is to continue implementation of SMI.		Accessed September 24, 2021 https://w5.salud.gob.sv/archivos/pdf/Informes-Labores-MSPAS-MINSAL-2004-

Doc No.	Author	Year	Title	Document Type	Brief description of the document.	Reviewer Initials (removed for peer review)	Notes<br>Also indicates most recent date accessed.
							2015/INFORME-LABORES-MINSAL-2014-2015_V2.pdf
15	Government of El Salvador	2015	El salvador productivo, educado y productivo. Plan quinquenal de desarrollo 2014-2019.	Plan	This development plan highlights health gains in previous five years. It mentions assistance to the 100 poorest municipalities. Notes that in some areas, up to 40% of workers may be paid below minimum wages. Security is a growing problem, where 20% of population claims to have been a victim of violence and direct costs may exceed 10% of GDP.<br><br>The document speaks of the health reform progress. The reform is based on unified, universal, free, equitable, participative, solidarity, and intersectoral principles. The 4th objective addresses health--gradual universal access and coverage to the El Salvadoran population. It sets four goals focused mostly on MCH: reduce MM to less than 35 per 100k live births, maintain child mort 8/1000, increase vaccination to 90-95, and reduce out of pocket costs by 3 percentage points.		MCH health identified as national priorities. Also, note that violence is a considerable problem.<br><br>Accessed October 26, 2021<br>https://observatorioplanificacion.cepal.org/sites/default/files/plan/files/ELSAL.pdf

Doc No.	Author	Year	Title	Document Type	Brief description of the document.	Reviewer Initials (removed for peer review)	Notes Also indicates most recent date accessed.
16	Ministry of Health of El Salvador	2015	Plan estrategico institucional en salud 2014-2019	Plan	This document lists laws, regulations, plans, and strategies relevant to the health strategy. It also provides a good summary of the health situation in the previous five years. It is aligned with the following policies: Política Nacional de Salud, el Sistema de Protección Social Universal (focus on health and education) y la estrategia de Comunidades Solidarias (100 poorest municipalities). It operationalizes the National Development Plan 2014-2019 and National Health Policy 2014-2019. It consists of five objectives, of which the second and third focus on holistic and integrated health services to the population. Objective 1 addresses the national health system which includes various entities such as military, social security, and education; objective 4 addresses health research and human resource development, and the last on strengthening MINSAL's oversight and regulatory role. The strategy is to be monitored monthly and evaluated every three months to generate information for decision-making.		The 100 poorest communities were part of a poverty alleviation program.<br><br>Accessed October 26, 2021 https://www.salud.gob.sv/download/plan-estrategico-institucional-en-salud-2014-2019/
17	Inter-American Development Bank	2015	Salud Mesoamerica 2015: Segunda operacion individual (ES-G1002). Propuesta de financiamiento no reembolsable.	Other	This document is the proposal for the second operation in El Salvador.		Accessed October 28, 2021 https://www.iadb.org/projects/document/EZSHARE-2022579177-20?project=ES-G1002

Doc No.	Author	Year	Title	Document Type	Brief description of the document.	Reviewer Initials (removed for peer review)	Notes Also indicates most recent date accessed.
18	Inter-American Development Bank	2015	Convenio individual de financiamiento no reembolsable de inversion del Fondo Mesoamericano de Salud entre la Republica de El Salvador y el Banco Interamericano de Desararollo en su calidad de administrador del Fondo Mesoamericano de Salud. Iniciativa Salud Mesoamerica - El Salvador. Segunda operacion individual.	Other	This document summarizes the accomplishments in the first operation and lays out the program for the second operation.		Accessed October 28, 2021 https://www.iadb.org/projects/document/EZSHARE-2056220512-943?project=ES-G1002;GRT/HE-14650-ES;GRT/HE-14651-ES
20	Ministry of Health of El Salvador	2016	Politica nacional de salud 2015-2019	Policy document	This policy highlights accomplishments in the past five years. It added two axes to the health reform: violence and environment. The document contains strategies for each of the ten axes, including human resources, to continue progress on health reforms. Recruiting staff, providing resources and training, and strengthening teams are included under the HR strategy.		The national health policy includes HR strenghtening.<br><br>Accessed October 26, 2021 https://rrhh.salud.gob.sv/node/109

Doc No.	Author	Year	Title	Document Type	Brief description of the document.	Reviewer Initials (removed for peer review)	Notes Also indicates most recent date accessed.
21	Ministry of Health of El Salvador	2016	Informe de labores 2015-2016	Report	This report mentions the final evaluation of human resources, as part of the Plan Decenal de Salud para las Américas, demonstrating gains since the 2010 baseline and 2012 midpoint evaluations. It also makes mention of El Salvador as a leader in the Region for identifying and measuring health inequities. The MOH doubled its health facilities from 413 in 2008 to 815 by 2016. 171 facilities were equipped with cell phones and internet, with all facilities planned to have the same by the end of 2016. The country has yet to achieve the goal of 25 human resources per 10,000 people (now at 18.4) but has increased (3x) resources in many areas. Importantly, a salary study was conducted this year to address salary inequities. The salary scales have not been modified since 1994 and the MOH recognizes the disparities that exist between the salary and the new demands of the health system (see p.153). SMI, along with other funders, provided 579 facilities with tablets that georeference the "fichas familiares". A total of 1800 tablets were funded (p. 137). A communication campaign was developed to communicate the SMI targets and 146 scholarships were awarded for the "diplomado latinoamericano AMANECE en salud materno-infantil."		According to this document, staff shortages still exist, although there have been some gains. Mention of a communication campaign to raise awareness about the SMI targets is relevant to our study.<br><br>Accessed September 24, 2021 https://w3.salud.gob.sv/download/informe-de-labores-2015-2016/

Doc No.	Author	Year	Title	Document Type	Brief description of the document.	Reviewer Initials (removed for peer review)	Notes Also indicates most recent date accessed.
22	Ministry of Health of El Salvador	2016	Lineamientos técnicos para la evaluación de resultados en salud en las RIISS	Guideline	This document provides monitoring and evaluation requirements for each SIBASI; prescriptive dates to meet every quarter; preparation of results and presentation meeting includes head nurses at all levels; team leaders; and regional and SIBASI directors; space created for reflexivity; requires improvement plan and followup with each level; tools provided as annexes for selecting indicators.		The SIBASI guidelines are relevant to our study with regards to quarterly meetings, who attends, space for reflexivity, and requirement of improvement plans.<br><br>Accessed December 23, 2017 http://asp.salud.gob.sv/regulacion/pdf/lineamientos/lineamientos_evaluacion_resultados_riiss.pdf
23	Ministry of Health of El Salvador	2017	Lineamientos Técnicos para el desarrollo de actividades en Ecos Familiares y Ecos Especializados (update)	Guideline	This is an update of the original guideline issued in 2011. In 2010, 100 teams were deployed in the 100 poorest communities. By 2014, there were 575 teams (ECOS) in 184 municipalities, based in basic or intermediate facilities (UCSFs). These were distributed according to the Mapa de Pobreza: Política Social y Focalización de El Salvador, FISDL/FLACSO. The guideline defines and describes the responsibilities of the two types of teams (PHC and Specialized PHC) as well as the process for their selection and deployment into the community. Rural teams had 600 families (3,000 people) and urban ones had 800 families (7,000 people). Importantly, it indicates the planning process for conducting the health risk assessment which involves mapping the community, holding round tables with different stakeholders, preparing and presenting an annual operations plan (POA), reviewing activities weekly with the team, and reviewing the POA every six months. Of note, one vehicle was to be shared with four teams, each using it one day a week.		SMI funded 75 of the 100 PHC teams deployed in the poorest communities. Of relevance is the description of team processes.<br><br>Accessed May 7, 2021 http://asp.salud.gob.sv/regulacion/pdf/lineamientos/lineamientos_tecnicos_para_los_Ecos_Familiares_y_Ecos_Especializados.pdf

Doc No.	Author	Year	Title	Document Type	Brief description of the document.	Reviewer Initials (removed for peer review)	Notes<br>Also indicates most recent date accessed.
24	Ministry of Health of El Salvador	2017	Informe de labores 2016-2017	Report	The report mentions the conclusion of the PRIDES loan program, that funded the SUIS (single health info system) including training of staff in its use and maintenance. It includes the budget expenditures for SMI. The MOH continued to implement SMI.		PRIDES ended in this time period.<br><br>Accessed September 24, 2021 https://w3.salud.gob.sv/download/informe-de-labores-2016-2017/
25	Ministry of Health of El Salvador	2017	MINSAL gana dos premios a la calidad y mejores practicas	Other	El Salvador government (through the Secretaría de Gobernabilidad y ES Calidad (award administrator) award program to ministries for best practices (Reconocimiento a las mejores practicas). MINSAL received two in 2017. It had won 5 in previous years for best practices that improved quality of care at various hospitals. MINSAL has submitted projects since 2012 when the award was created.		This is a rare example of public recognition in the public sector.<br><br>Accessed December 26, 2017 http://www.salud.gob.sv/12-12-2017-minsal-gana-dos-premios-a-la-calidad-y-las-mejores-practicas/
29	Ministry of Health of El Salvador	2018	Informe de labores 2017-2018	Report	Noted that facilities had distributed 1878 tablets with plans to distribute a total of 2782 by the end of the year. The MOH continued to implement SMI.		Accessed September 24, 2021 https://w3.salud.gob.sv/download/informe-de-labores-2017-2018/
30	Inter-American Development Bank	2018	Convenio individual de financiamiento no reembolsable de inversion del Fondo Mesoamericano de Salud entre la Republica de El Salvador y el Banco Interamericano de Desararollo en su calidad de administrador del	Other	This agreement lays out the third operation of SMI in El Salvador. It provides a summary of accomplishments in the second operation.		Accessed October 28, 2021 https://www.iadb.org/projects/document/EZSHARE-2056220512-6307?project=ES-G1003

Doc No.	Author	Year	Title	Document Type	Brief description of the document.	Reviewer Initials (removed for peer review)	Notes<br>Also indicates most recent date accessed.
			Fondo Mesoamericano de Salud. Iniciativa Salud Mesoamericana - El Salvador. Tercera operacion individual.				
35	Ministry of Health of El Salvador	2019	Informe de labores 2018-2019	Report	The report mentions funding the construction and equipping of a new facility with the second tranche. The MOH continued to implement SMI.		Accessed September 24, 2021<br>https://w3.salud.gob.sv/download/informe-de-labores-2018-2019/
36	Ministry of Health of El Salvador	2019	Reforma de salud: más allá de los servicios de salud. Congreso de la reforma de salud de El Salvador.	Report	Provides a history of the evolution of the health reforms in El Salvador; highlights by decade.<br>Prior to 2009 reform, privatization was favored in reforms; during the war the health system deteriorated to a point where rural populations did not have access to health services and facilities were in poor conditions; most health professionals were concentrated in urban areas; social organizations, including professional associations protested privatization and advocated for an equitable system. Health promoters were first introduced in rebel territory to provide basic health services. Community selected and trained them. The leftist government consolidated previous efforts of moving the health system to a rights-based system with universal coverage and access based on PHC principles as proposed in Alma Ata.<br><br>This report was turned into a publication. See Transformación de la salud en El Salvador Diez años de reforma hacia la salud universal.		Provides background information on the context before the health reform.<br><br>Accessed October 26, 2021<br>https://rrhh.salud.gob.sv/files/webfiles/Libro-Reforma-de-Salud-mas-alla-de-los-servicios-de-salud-congreso-de-la-reforma-de-salud-de-el-salvador-v2.pdf

Doc No.	Author	Year	Title	Document Type	Brief description of the document.	Reviewer Initials (removed for peer review)	Notes Also indicates most recent date accessed.
40	Ministry of Health of El Salvador	2020	Memoria de labores 2019-2020	Report	A new government came into power in June 2019. It continued to make chidren and women's health a priority. The MOH received the first payment for the 3rd operation.		Accessed September 24, 2021 https://w3.salud.gob.sv/download/memoria-de-labores-2019-2020-junio-2019-a-mayo-2020/
41	Ministry of Health of El Salvador	2021	Memorias de labores 2020-2021	Report	The MOH continued to implement SMI and support the 14 SMI municipalities. Funding for the third operation continued to support maternal and child health and strengthening of support systems and intersectoral strategies. The report mentions that all health workers received raises. Additional health promoters were hired.		Accessed October 26, 2021 https://www.transparencia.gob.sv/institutions/minsal/documents/memorias-de-labores
