# Supplementary material for: Characterizing the implementation of performance management interventions in a primary health care system: a case study of the Salud Mesoamerica Initiative in El Salvador
Source: Health Policy Plan. 2023 Mar 27;38(5):579–92. doi: 10.1093/heapol/czad020 (PMC10190960; doi:10.1093/heapol/czad020)
Supplement: czad020_Supp [file czad020_supp.zip › S-3 Codebook.pdf]

Supplementary File S-3 Codebook

PHC Performance Management -- El Salvador Case Study Codebook									
**General Instructions:**	**Select one question and the full response as a coding unit to assign a code or codes. See notes below from Update_17Feb21 for further instructions selecting questions.**		Date created: December 4, 2020<br>Date modified: January 26, 2021, February 17, 2021, March 22, 2021						

Parent Code	Description	Child Code	Description	Grandchild Code	Description	Exclusion Criteria	Notes	Inductive	Deductive
**1. Characteristic of individuals**	Refers to personal attributes about the actors involved with the intervention.	1.1 Knowledge & Beliefs about the Intervention	Individuals' attitudes toward and value placed on the intervention as well as familiarity with facts, truths, and principles related to the multi-faceted intervention (i.e., target setting, performance measurement, feedback, and in-kind incentives). Use this code when the respondent speaks about SMI/Mesoamerica in general terms. Code to a specific intervention code when the interviewer asks specifically about one of the four interventions, or the respondent mentions these.						✓
		1.2 Self-efficacy	Individual belief in their own capabilities to execute courses of action to achieve implementation targets.						✓

Parent Code	Description	Child Code	Description	Grandchild Code	Description	Exclusion Criteria	Notes	Inductive	Deductive
		1.3 Individual Stage of Change	Characterization of the phase an individual is in, as he or she progresses toward skilled, enthusiastic, and sustained use of the intervention.				We may end up dropping this code. The interview guide did not include specific questions about this.		✓
		1.4 Individual Identification with Organization	A broad construct related to how individuals perceive the organization, and their relationship and degree of commitment with that organization (i.e., PHC facility or PHC system), and its goals. Apply this code when the respondent mentions relating to the values of SMI and his or her commitment to implementing health care services to meet the targets.				Also, code for compatibility.		✓
		1.5 Other Personal Attributes	A broad construct to include other personal traits such as tolerance of ambiguity, intellectual ability, motivation, values, competence, capacity, and learning style. Code work motivation separately. See below under mechanisms. Also apply this code when the respondent mentions his/her professional and personal experience.						✓
**2. Outer setting**	Refers to the context outside of the team organizational context.	2.1 Patient Needs & Resources	The extent to which patient needs, as well as barriers and facilitators to meet those needs, are accurately known and						✓

Parent Code	Description	Child Code	Description	Grandchild Code	Description	Exclusion Criteria	Notes	Inductive	Deductive
			prioritized by the organization.						
		2.2 Cosmopolitanism	The degree to which an organization is networked with other external organizations. These can be local, regional, national, or international organizations.						✓
		2.3 Peer Pressure	Mimetic or competitive pressure to implement an intervention; typically, because most or other key peer (e.g., other PHC facilities) or competing organizations have already implemented or are in a bid for a competitive edge.				We may end up dropping this code.		✓
		2.4 External Policy & Incentives	A broad construct that includes external strategies to spread interventions, including policy and regulations (governmental or other central entity), external mandates, recommendations and guidelines, pay-for-performance, collaboratives, and public or benchmark reporting. Code target setting, performance measurement, feedback, and in-kind incentives separately. See below under intervention approaches. Apply this code when the respondent mentions the Ministry of Health or specific policies.						✓

Parent Code	Description	Child Code	Description	Grandchild Code	Description	Exclusion Criteria	Notes	Inductive	Deductive
**3. Inner Setting**	Refers to the team organizational context.	3.1 Structural Characteristics	The social architecture, age, maturity, and size of an organization.						✓
		3.2 Networks & Communications	The nature and quality of webs of social networks and the nature and quality of formal and informal communications within an organization. Apply the code cosmopolitism (2.2) when the respondent mentions networking or communicating with others outside the organization.						✓
		3.3 Culture	Behavioral norms, values, and basic assumptions of a given organization reflected in the behavior of team members or leadership in their interaction with each other. Also apply this code when the respondent mentions how personnel matters are addressed within the organization or team.						✓
		3.4 Implementation Climate	The absorptive capacity for change, shared receptivity of involved individuals to an intervention, and the extent to which use of that intervention will be rewarded, supported, and expected within their organization.	*3.4.1 Tension for Change*	The degree to which stakeholders (i.e., team members and MoH officials) perceive the current situation as intolerable or needing change.				✓

Parent Code	Description	Child Code	Description	Grandchild Code	Description	Exclusion Criteria	Notes	Inductive	Deductive
				3.4.2 Compatibility	The degree of tangible fit between meaning and values attached to the intervention by involved individuals, how those align with individuals' own norms, values, and perceived risks and needs, and how the intervention fits with existing workflows and systems.				✓
				3.4.3 Relative Priority	Individuals' shared perception of the importance of the implementation within the organization. For example, the respondent mentions focusing more on services that are being measured than prior to SMI.		"we pay greater attention, I would say, to the areas of children, women, puerperal women, and specially to reduce the death rate in our district"		✓
				3.4.4 Organizational Incentives & Rewards	Extrinsic incentives such as goal-sharing awards, performance reviews, promotions, and raises in salary, and less tangible incentives such as increased stature or respect. Code in-kind incentives separately. See material rewards below under intervention approaches.				✓
				3.4.5 Goals and Feedback	The degree to which goals are clearly communicated, acted upon, and fed back to staff, and alignment of that feedback with goals. [Note there may be overlap with team feedback under intervention approaches and reflecting and evaluating under implementation process. Apply this code when the respondent mentions team targets or other organizational goals.				✓

Parent Code	Description	Child Code	Description	Grandchild Code	Description	Exclusion Criteria	Notes	Inductive	Deductive
				3.4.6 Learning Climate	A climate in which: a) leaders express their own fallibility and need for team members' assistance and input; b) team members feel that they are essential, valued, and knowledgeable partners in the change process; c) individuals feel psychologically safe to try new methods; and d) there is sufficient time and space for reflective thinking and evaluation. Code quantitative and qualitative feedback separately. See below under intervention approaches. Also apply reflecting and evaluating (5.4) when the respondent mentions using team feedback/evaluation results.				✓
		3.5 Readiness for Implementation	Tangible and immediate indicators of organizational commitment to its decision to implement an intervention.	*3.5.1 Leadership Engagement*	Commitment, involvement, and accountability of leaders and managers (e.g., team leaders) with the implementation. There may be overlap with engaging under implementation process. Use this code when there is mention of commitment, involvement, and accountability of leaders in the MoH and facility.				✓
				3.5.2 Available Resources	The level of resources dedicated for implementation and on-going operations, including money, training, education, physical space, and time.				✓
				3.5.3 Access to Knowledge & Information	Ease of access to digestible information and knowledge about the intervention and how to incorporate it into work tasks. Code team feedback separately, see under intervention approaches.				✓

Parent Code	Description	Child Code	Description	Grandchild Code	Description	Exclusion Criteria	Notes	Inductive	Deductive
**4. Intervention Approaches**	Refers to strategies used to implement the intervention(s). Note: Although respondents may mention these, descriptions of each and how they are intended to work will likely be found in the documents to be reviewed.	4.1 External Verification	Independent verification by SMI experts of the achievement of team-based performance targets, carried out every six months. Facility surveys and household surveys were conducted to collect the data. Apply this code when respondents mention this implementation approach. Use this code when the respondent mentions external assessment or evaluation.						✓
		4.2 Team Feedback	Quantitative and qualitative feedback about the team performance from SMI measurement. Quantitative feedback can be in the form of reports or tables, for example. Qualitative feedback may be conveyed verbally in a team setting or individually by a supervisor or MoH official. Apply this code when respondents mention this implementation approach.						✓
		4.3 Material Rewards	In-kind incentives linked to the achievement of targets. Apply this code when respondents mention this implementation approach.						

Parent Code	Description	Child Code	Description	Grandchild Code	Description	Exclusion Criteria	Notes	Inductive	Deductive
		4.4 Public Recognition	Team recognition for good performance given in a public meeting. SMI provided certificates to recognize good performance (i.e., obtaining 60 or more points on a 100-point scale). Apply this code when respondents mention this implementation approach.						✓
**5. Implementation Process**	Refers to the change process aimed to achieve individual and organizational level use of the intervention as designed. It consists of interrelated, non-sequentially occurring subprocesses that may occur simultaneously at the team level.	5.1 Planning	The degree to which a scheme or method of behavior and tasks for implementing an intervention are developed in advance, and the quality of those schemes or methods. Apply this code when the respondent mentions meetings or attending meetings where new strategies to meet targets are discussed.						✓
		5.2 Engaging	Attracting and involving appropriate individuals in the implementation and use of the intervention through a combined strategy of social marketing, education, role modeling, training, and other similar activities. Individuals may be opinion leaders who may influence the attitudes and beliefs of their colleagues; individuals appointed with the responsibility for implementing the intervention such as team leaders; champions who support an						✓

Parent Code	Description	Child Code	Description	Grandchild Code	Description	Exclusion Criteria	Notes	Inductive	Deductive
			intervention(s) overcoming indifference or resistance that the intervention may provoke in an organization; or external change agents who influence or facilitate intervention decisions in a desirable direction, for example.						
		5.3 Executing	Carrying out or accomplishing the implementation according to plan.						✓
		5.4 Reflecting & Evaluating	Quantitative and qualitative feedback about the progress and quality of implementation accompanied with regular personal and team debriefing about progress and experience. Use this code when there is mention of team meetings to discuss feedback.						✓
**6. Causal Mechanisms**	Refers to the actions and reactions, or behaviors, triggered among program actors by the interactions between the context and SMI's interventions.	6.1 Goal Alignment	Linking individual goal outcomes with organizational goal outcomes.						✓

Parent Code	Description	Child Code	Description	Grandchild Code	Description	Exclusion Criteria	Notes	Inductive	Deductive
		6.2 Work Motivation	An individual's degree of willingness to exert and maintain an effort towards organizational goals. Code as an outcome at the individual level if this construct is discussed as an effect of the intervention. See outcome code under proximal outcomes below.				Need to distinguish between mechanism and outcome.		✓
		6.3 Collective Efficacy	Shared belief in the team's ability to organize and complete interdependent tasks to meet targets.						✓
		6.4 Community Recognition	Apply this code when the respondent mentions receiving acknowledgement from community members for providing services despite travel difficulties or security challenges, for example. Acknowledgement may be verbal, as in being thanked, or through offers of a meal or a food treat or being invited to family or community events as a form of showing appreciation and gratitude towards the respondent or community health team.					✓	

Parent Code	Description	Child Code	Description	Grandchild Code	Description	Exclusion Criteria	Notes	Inductive	Deductive
**7. Proximal Outcomes (Individual)**	Refers to immediate positive or negative changes produced by an intervention – directly or indirectly, intended, or unintended – at the individual level.	7.1 Job Satisfaction	Refers to emotional reactions and thoughts about work, resulting from comparing actual with expected performance outcomes. Refers to respondents mentioning of how happy they are with their job and accomplishments. Also apply this code when the respondent mentions that they feel valued, important, or respected.				There is no one agreed upon definition for job satisfaction. Locke E. A. & Henne D.C. (1986) focused on the emotional aspect.		✓
		7.2 Morale	Refers to the attitude and feeling towards work affected by the organizational environment. It can be high when there is an enabling environment or low when the environment is not conducive to a positive work experience.				There is no one agreed upon definition in the literature about morale. It is a multi-dimensional construct sometimes measured by the following indicators: job satisfaction, work motivation, and burnout.		✓
		7.3 Work Motivation	Increase or decrease in willingness to exert and maintain an effort towards organizational goals. Code as mechanism if this construct is discussed as an action or reaction, or behavior, triggered by the interaction of the context and the intervention. See code for mechanism under causal mechanisms above.						✓

Parent Code	Description	Child Code	Description	Grandchild Code	Description	Exclusion Criteria	Notes	Inductive	Deductive
**8. Proximal Outcomes (Team)**	Refers to immediate positive or negative changes produced by an intervention – directly or indirectly, intended, or unintended – at the team level.	8.1 Adoption and Adaptation of PM Interventions	Refers to respondents mentioning the team's use of PM interventions and modifying them as needed to support their daily work. [NOTE: Only adoption may apply to this case study. The teams are not changing (adapting) the interventions.]						✓
		8.2 Changes in Service Delivery	Refers to respondents mention of changes the team has made to the outreach and facility services provided based on the needs of the community and to meet targets.						✓
		8.3 Cohesiveness	Refers to the social bonds team members have developed, and the extent team members are united in accomplishing tasks to meet targets.				There is no one agreed upon definition in the literature about team cohesion or cohesiveness. This is a multi-dimensional construct.		✓
		8.4 Commitment to Targets	Refers to respondents mentioning commitment as a team to accomplishing targets.						✓
		8.5 Commitment to Community	Refers to respondents mentioning commitment of the team to deliver PHC services to the community and improving the overall health of the community.						✓

Parent Code	Description	Child Code	Description	Grandchild Code	Description	Exclusion Criteria	Notes	Inductive	Deductive
**Inductive Codes--new category to be added**	New codes may emerge from the data. Add new categories here. If a code expands an existing category, add it above in the appropriate deductive category and indicate that it is an inductive code with a check mark under the inductive column.								
								✓	
								✓	
								✓	
