# Supplementary material for: Characterizing the implementation of performance management interventions in a primary health care system: a case study of the Salud Mesoamerica Initiative in El Salvador
Source: Health Policy Plan. 2023 Mar 27;38(5):579–92. doi: 10.1093/heapol/czad020 (PMC10190960; doi:10.1093/heapol/czad020)
Supplement: czad020_Supp [file czad020_supp.zip › S-4 Code summary memo template.pdf]

Project Name: El Salvador PM Case Study | Code name: | Version date: 9 *April 2020*

Supplementary File S-4: Code summary memo template
Code Summary: [Insert Code Name]
El Salvador PM Case Study

Instructions: *A Code summary is a compilation of all data collected from across respondents on a particular coded node. The purpose of a code summary is to analyze all data from a specific code and organize it in a coherent way for publication of the results. These summaries are created only after coding is completed. Code summaries should be created by reading all data extracts coded with the code of interest, identifying the major and contradictory ideas, and summarized using the headings and domains provided below:*

1. **Code**: [insert code name that matches with NVivo file code name, followed by the code number in parenthesis] e.g., Material Rewards (4.3)
2. **Stakeholder(s)**: [list stakeholders represented in this coding summary] e.g., Providers, MoH officials, SMI experts; (n=X)
3. **Code summary created by:** [Enter your name]
4. **Version date:** [insert date of current version of code summary in Day, Month Year format] e.g., 9 April 2020
5. **Code definition**: Copy and paste the title and definition of the code you are summarizing from the PM codebook (see link): [insert link]

    a. EXAMPLE: **Material Rewards:** In-kind incentives linked to the achievement of targets. Apply this code when respondents mention this implementation approach.

6. **Code summary Table:**

**Respondent ID** *[List respondent ID for all included references in the query]*	**Demographics** *[List any relevant demographics as provided. This is useful for preparation for publication quotes. Note: Put N/A if unavailable]*	**Brief points catching important aspects** *[Briefly mention key points of interest from this respondent's references. Can include their stakeholder status, number of years with the PHC Team or MoH , a summary point from their narrative, whether their points align with or deviate from the main narrative, etc.]*
[add as many rows as necessary]	Age:<br>Gender:<br>Occupation:<br>[Other]: *[Include any other available SES indicators of interest for the study e.g., education.]*	

7. **Main narrative(s) (n=x; Respondent IDs)**: *[In this section, summarize in 2-3 paragraphs the major narrative(s) for this code for this stakeholder group(s). Indicate the number of respondents and respondent IDs of those who endorsed this dominant view. GIVE YOUR MAIN NARRATIVE A TITLE]*

Key quotes: *[Include 2-4 quotes from participants that are (1) exemplary of this major narrative; or (2) are "catchy": i.e. can be used in a slogan/title or captures the essence of the narrative in a creative, expressive, or meaningful way.]*

8. **Connections to other codes**: *[If there are connections to other codes, then include 1) any relevant code names; 2) a brief description of the connection and 3) supporting quotes.]*

9. **Deviant narratives (n=x; Respondent IDs)**: *[Include narrative(s) from respondents that are either contrasting from the main narrative(s) or offer alternative perspectives. Include the number of participants and respondent IDs for deviant narratives. If applicable, indicate any potential explanatory factors that may be causing the deviant narrative (identified via additional analysis, or drawing from literature, or researcher speculation). GIVE YOUR DEVIANT NARRATIVE A TITLE]*

Key quotes: *[Include quotes to capture all deviant perspectives. Absolutely include any quote(s) that directly contradicts the dominant narrative.]*

10. **Code Query Output** *[Copy and paste the entire code query output at the bottom of the Code Summary]*
